# Foliar application of green-synthesized MoO₃ nanoparticles alleviates salinity stress in molokhia by boosting antioxidant defense and stress-related gene expression

**DOI:** 10.1186/s12870-026-09007-6

**Published:** 2026-05-26

**Authors:** Doaa E. Elsherif, Osama A. Alaziz, Eman H. Rashed, Aya M. Hewidy, Roqia A. Abdelazim, Salma A. Dabash, Mai A. El-Esawy

**Affiliations:** 1 Botany Department, Faculty of Science, Tanta University, Tanta, 31527 Egypt; 2 Biotechnology Department, Faculty of Science, Mansoura University, Mansoura, 35516 Egypt; 3 Zoology Department, Faculty of Science, Kafrelsheikh University, Kafrelsheikh, 33516 Egypt; 4 Chemistry and Microbiology Department, Faculty of Science, Menoufia University, Menofia, 32511 Egypt; 5 Zoology Department, Faculty of Science, Menoufia University, Shebin El-Kom, 32511 Egypt

**Keywords:** Salinity, Biogenic nanoparticles, Molybdenum trioxide, Antioxidants, Gene expression, *Corchorus olitorius* L

## Abstract

**Background:**

Salinity stress is a major global challenge that impairs plant growth by disrupting numerous physiological and biochemical processes. Nano-fertilizers, particularly green synthesized nanoparticles, offer a promising and eco-friendly strategy to enhance plant resilience and productivity under such stress. Accordingly, this study addresses a significant research gap by exploring the potential role of Molybdenum trioxide nanoparticles (MoO₃NPs) in improving salinity tolerance in molokhia, a topic that has received limited attention in previous studies.

**Results:**

Molybdenum trioxide nanoparticles (MoO₃NPs) were fabricated using *Medicago polymorpha* fruit extract and characterized as spherical, orthorhombic α-MoO₃ with an average size of 9.2 ± 2.7 nm. Molokhia (*Corchorus olitorius* L.) was foliar-sprayed with MoO₃NPs at various dosages (0, 25, 50, and 100 mg/L) to mitigate the harmful effects of salinity stress (250 mM NaCl) on 30-day-old Molokhia plants. Application of 250 mM NaCl significantly inhibited growth, photosynthetic pigments, antioxidant activity, and osmolyte accumulation, alongside increased oxidative stress markers (MDA and H_2_O_2_). Foliar spraying with biogenic MoO_3_NPs under saline conditions, especially at 50 mg/L, effectively mitigated salt-induced damage by enhancing growth parameters, restoring photosynthetic pigment levels, and improving osmotic adjustment by boosting proline and soluble protein levels compared with control. Moreover, MoO_3_NPs treatment elevated enzymatic and non-enzymatic antioxidants (phenolics, flavonoids, glutathione, and ascorbic acid) and upregulated key stress-responsive genes (*phenylalanine (PAL), magnesium chelatase (CHLH), superoxide dismutase1 (SOD1), catalase2 (CAT2),* and *flavonol synthase (FLS)*), thereby reducing oxidative stress markers. These effects mitigated ROS accumulation, improved cellular stability, and enhanced overall plant resilience.

**Conclusion:**

These findings highlight that green-synthesized MoO₃NPs act as efficient nanofertilizers that alleviate salinity stress by promoting growth, reinforcing antioxidant defenses, and modulating stress-related genes. This dual role not only enhances crop resilience but also enriches the medicinal and nutritional value of Molokhia.

## Background

As climate change continues to alter global ecosystems, understanding and addressing plant salinity stress becomes crucial for sustaining agriculture and ensuring food production in affected regions [1, 2]. Salinity stress, a pervasive environmental challenge, significantly impedes plant growth and productivity, posing a substantial threat to global agriculture As a result of salinity, reactive oxygen species (ROS) increase and create oxidative stress in plant cells, which damages lipid synthesis and membrane stability, as well as DNAand proteins [3, 4]. Salt concentrations above the threshold of a plant's ability to absorb water, assimilate nutrients, and capture sunlight reduce yields and even result in plant death when excessive salt concentrations are present [5]. Plants employ a suite of adaptive mechanisms to cope with salinity, ranging from ion exclusion and compartmentalization to osmoprotectants (proline, glycine betain, sugars, and amino acids) and activation of antioxidant defense systems like, non-enzymatic and enzymatic antioxidants [6, 7]. To ensure tolerance to salinity stress, the plant enhances the activity of numerous transcription factors, genes, and metabolites. A comprehensive understanding of these physiological responses is crucial for developing effective strategies to safeguard food security in saline-affected regions [8, 9].

Nanotechnology has emerged as a viable strategy to alleviate salt stress in agriculture, which increasingly jeopardizes global food security [10, 11]. Salt stress diminishes agricultural yield and quality by interfering with physiological and biochemical processes in plants [12]. Nanomaterials (NMs) have demonstrated potential in improving plant stress tolerance by safeguarding photosynthesis, mitigating oxidative stress, and preserving osmotic and ionic equilibrium [13, 14]. NMs additionally facilitate seed germination, plant development, disease resistance, genetic modification, and agrochemical detection [15, 16]. The use of nanotechnology in plant science offers an economical and sustainable method for enhancing agricultural productivity. Additional investigations into the molecular and phytohormonal mechanisms of nano-enabled plant stress tolerance may facilitate the formulation of viable strategies to mitigate salt stress in crops [10].

A promising biotechnological trend is the green synthesis of nanoparticles (NPs) smaller than 100 nm [17, 18]. Compared with conventional physical and chemical fabrication methods, this biofabrication method is environmentally friendly, non-toxic, efficient, and more economical [19, 20]. Metal oxide nanoparticles offer greater chemical and thermal stability than elemental nanoparticles, reducing the risks of oxidation, degradation, and toxicity in plant systems [21]. Their unique optical, electrical, and catalytic properties enhance photosynthesis, antioxidant activity, and stress tolerance. Moreover, their stability and low solubility minimize environmental contamination risks, making them safer for sustainable agricultural use [22, 23]. The plant-based green synthesis of nanofertilizers like Molybdenum trioxide is garnering more focus at present because it is simple, economical, trustworthy, and environmentally friendly [24]. The bioactive compounds in plant extracts are crucial for stabilizing MoO₃ nanoparticles (NPs), as they possess both reducing and stabilizing properties [25]. Molybdenum is an essential micronutrient that plays a pivotal role in plant metabolism, primarily serving as a cofactor for crucial enzymes, including nitrate reductase, xanthine dehydrogenase, and sulfite oxidase. These enzymes are integral to nitrogen assimilation, redox homeostasis, carbon metabolism and detoxification processes [26, 27]. These processes are essential for plants to thrive under varying environmental conditions. Although biologically inactive on its own, molybdenum deficiency impairs these enzymes, particularly those crucial for nitrogen assimilation, leading to growth defects in plants [26].

The genus Corchorus (family Tiliaceae commonly Malvaceae) includes an estimated 40–100 species of flowering plants, with *C. olitorius* and *C. capsularis* being the primary cultivated varieties [28]. Molokhia (*Corchorus olitorius* L.), a leafy green vegetable with medicinal properties, is extensively cultivated in tropical and subtropical regions. In Egypt, it is primarily available during the spring and summer months [29]. Molokhia leaves have been Egyptian staple foods since the time of pharaohs. Molokhia contains significant levels of proteins, iron, calcium, and vitamins (A, C, and E) [30]. Moreover, Molokhia is highly nutritious, and contains valuable compounds such as vitamins, carotenoids, phenolics, tannins, and glycosides [31, 32]. Numerous phytochemical compounds including corchorin, corchorgenin, cardiac glycosides, corchortoxin, and capsularin, corchoritin, and olitoriside have been identified in both the leaves and seeds of Molokhia [33]. Despite its considerable economic value, Molokhia cultivation is susceptible to salinity stress, which adversely affects seed germination, vegetative growth, fiber quality, and overall yield. With the progressive salinization of arable lands, enhancing salinity tolerance in Molokhia plant has become a critical agronomic priority [34]. Consequently, the exploration of innovative strategies, such as the application of metal oxide nanoparticles to improve stress tolerance, is both timely and imperative. Although metal oxide nanoparticles such as TiO₂, ZnO, CuO, and Fe₃O₄ have shown promise in alleviating salinity stress in crops like wheat, tomato, and maize, respectively, their application remains untested in molokhia, a salinity-vulnerable leafy green staple in arid regions like Egypt [35–37]. Moreover, biofabricated MoO_3_NPs for eco-friendly stability, have not been investigated for salinity tolerance in any plant species, despite molybdenum's essential role in nitrogen assimilation and stress enzymes. Thus, this work is the first to evaluate biofabricated MoO₃ NPs' efficacy in enhancing molokhia's physiological, biochemical, and molecular responses under salinity, addressing a critical gap in nano-agriculture for underrepresented crops.

## Materials and methods

### Preparation of plant extract

The synthesis of MoO₃NPs employed a fruit extract from *Medicago polymorpha* as a green reducing agent. The plant material was obtained from Kafr Abu Jundi, Qutur, Gharbia Governorate, Egypt (30° 59′ 31″ N, 31° 00′ 14″ E). The plants were formally identified by Dr. Esraa E. Ammar, lecturer at Tanta University Herbarium (TANE), Egypt. Herbarium specimens were deposited in Tanta University Herbarium (TANE) with Acc. NO. 13,493. The fresh fruits of *M. polymorpha* were thoroughly washed with tap water and distilled water to remove any debris. To prepare the extract, 10 g of the whole fruits were boiled in 100 mL of deionized water at 60 °C for 30 min, and then filtering and centrifuging (4000 rpm, 15 min) the cooled solution to obtain the supernatant for synthesis. The obtained supernatant was utilized directly for the synthesis without further dilution (crude extract 10% (w/v)) to ensure a high concentration of reducing and stabilizing agents.

### Green synthesis of MoO_3_NPs

**Fig. 1:**
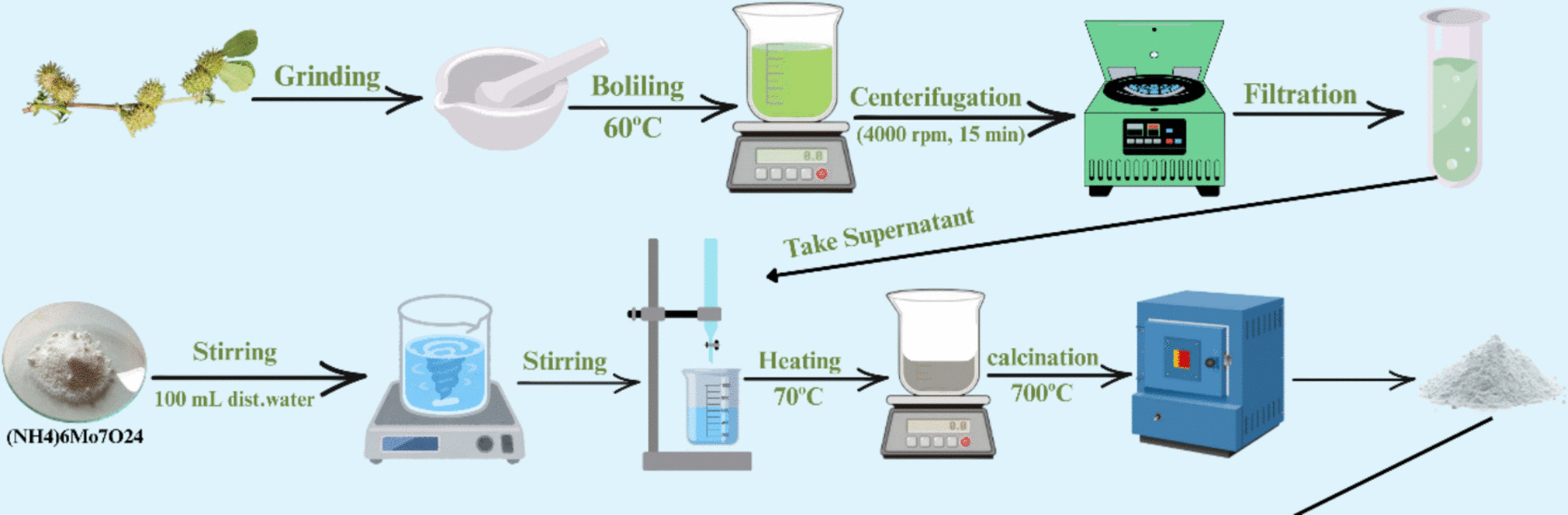
Schematic representation of the green synthesis process of MoO_3_ nanoparticles

The synthesis of the MoO_3_NPs involved preparing a solution of ammonium heptamolybdate (0.5 mM) by dissolving it in distilled water (100 mL) under 15 min of stirring (500 rpm) at room temperature (25 ± 2 °C). Following this, 5 mL of an aqueous extract from *M. polymorpha* fruit was introduced dropwise into the solution under constant vigorous stirring (500 rpm) at temperature of 40 °C to facilitate the synthesis of MoO₃NPs [38]. Heating the solution to 70 °C caused the supernatant to fully evaporate. The dry residue at the end was of a dark grayish hue. The resulting product was subjected to calcination for two hours at 700 °C at a controlled heating rate of 5 °C/min using a muffle furnace [38]. As calcination proceeded, the product's color gradually changed from dark grayish brown to gray (Fig. 1).

### Characterization of MoO_3_NPs

Preliminary validation of MoO_3_NP production was evaluated utilizing a UV–visible spectrophotometer (Shimadzu, Kyoto, Japan; Dual Beam Spectrophotometer, UV-1800). The investigation utilizing Fourier-transform infrared (FTIR) spectroscopy was conducted with a Perkin-Elmer 1430 infrared spectrophotometer, USA; that features an IR Affinity-1 model. In addition, the morphological properties of MoO_3_NPs were analyzed using transmission electron microscopy (TEM). A limited quantity of nanoparticle dispersion was deposited onto a carbon-coated copper grid to produce thin films of MoO_3_NPs. The samples underwent drying in a vacuum desiccator and were subsequently analyzed using a TEM microscope (JEOL JEM 1400; Japan). After that, X-ray diffractometer (Bruker Co.D8 Discover, Cu target, Wavelength 1.54A, 40 kV 40 mA, Germany) were implemented.

### Plant materials and experimental design

Seeds of Molokhia (*Corchorus olitorius* L.) (Falahy) were sourced from the Gemmeiza Agriculture Research Station in Egypt. For the investigation, seeds were surface-sterilized with 1% sodium hypochlorite for 10 min. The five seeds were sown per pot containing 5 kg of an air-dried soil mixture (sand:clay, 2:1 v/v). The physicochemical properties of the soil used revealed the following concentrations: N = 0.7, *P* = 2.4, K = 34.3, Ca = 5.6, Mg = 23.2, Na = 6.1, and Cl = 6.69 mg/kg. The soil had a slightly alkaline pH of 7.1 and an electrical conductivity (EC) of 3.5 mS/cm. The experiment was carried out in 1–30 June at the experimental garden of the Faculty of Science, Tanta University, Egypt. The prevailing environmental conditions corresponded to typical Egyptian summer climate, characterized by elevated temperatures, with mean daily values ranging from 33 ± 2 °C, relative humidity levels of approximately 50–60%, and an extended photoperiod exceeding 13–14 h of daylight. The pots were irrigated daily with tap water until full germination occurred. On the seventh day after sowing, germinated seedlings were divided into two primary salinity groups: a control (0 mM NaCl) and a salt-stressed group (250 mM NaCl). The salinity treatment was applied gradually over three days to avoid osmotic shock. The salt-stressed group was further subdivided into four treatment groups that received foliar sprays of biogenic MoO₃NPs twice weekly at concentrations of 0, 25, 50, and 100 mg/L. The foliar spray was applied using a manual pressure sprayer during early morning hours, when wind speed was minimal (< 5 km/h) and relative humidity was at its peak. The total volume of MoO₃NPs solution applied was 5 mL per plant for each foliar spraying. The experiment was arranged in a completely randomized design with three replicates. Plants were harvested after 30 days of growth for subsequent analysis.

### Growth parameters

The shoot length, root length, fresh weight and dry weight were measured for estimating the Molokhia growth performance.

### Measurement of photosynthetic pigments

Fresh tissue (50–100 mg fresh weight) from the growing Molokhia leaves was sampled for chlorophyll and carotenoid quantification via a spectrophotometer after extraction in 80% acetone, following [39] method. The absorbance was measured at 663, 644, and 480 nm, which allowing us to estimate the content of photosynthetic pigments.

### Osmolytes contents

Total soluble sugars were measured according to the phenol–sulfuric acid method of [40]. Using a spectrophotometer (V-1200), absorbance was read at 490 nm and the content was determined as mg per gram of dry weight.

Total soluble proteins were measured according to the Bradford [41] assay. Spectrophotometric absorbance was read at 595 nm, and the protein content, determined via a BSA standard curve, was expressed in mg per gram dry weight.

The proline content was measured using the protocol established by [42]. Using a spectrophotometer (model V-1200), readings were taken at 520 nm. The concentration was then calculated by comparing against a proline standard curve and is reported as mg per gram of dry weight.

### Oxidative stress markers

Malondialdehyde (MDA) content was determined spectrophotometrically by the thiobarbituric acid (TBA) method [43]. Measurements at 532 and 600 nm were used to calculate concentration (ε = 155 mM⁻^1^·cm⁻^1^), expressed as µmol/g fresh weight.

Following the protocol established by [44], we determined the concentration of hydrogen peroxide (H₂O₂) in fresh leaf tissue. The H_2_O_2_ content was directly estimated at 390 depending on spectrophotometer (V-1200).

### Non-enzymatic antioxidants

To measure total antioxidant capacity (TAC), we followed the phosphomolybdenum method of [45]. Mixing 0.6 M sulfuric acid, 4 mM ammonium molybdate, and 28 mM sodium phosphate in equal parts (1:1:1). Adding the methanolic extract to the reagent and boiling the mixture for 90 min. After cooling, the absorbance of the samples was measured at 765 nm.

The method of [46] was used to estimate ascorbic acid (ASA) levels. Following extraction of fresh leaves in 5% sulfosalicylic acid, the extract was added to a reaction mixture of 2% sodium molybdate, 0.15 N sulfuric acid, and 1.5 mM disodium phosphate. This mixture was heated to 60 °C for 45 min, then cooled, centrifuged, and analyzed spectrophotometrically at 660 nm. Results were calculated as mg per g fresh weight.

Following the protocol of [47], reduced glutathione (GSH) levels were determined spectrophotometrically at 412 nm using DTNB (5,5'-dithio-bis(2-nitrobenzoic acid)) as the assay reagent.

Total phenolic content was quantified using the Folin-Ciocalteu reagent according to the method of [48]. A spectrophotometer was used to assess absorbance at 650 nm, and the content was calculated based on a gallic acid standard curve.

The total flavonoid content was assayed by a method recorded by [49]. At 417 nm, the absorbance was measured and contrasted with a quercetin curve standard.

### Enzymatic antioxidants

The enzymatic antioxidants were extracted according to a method described by [50], homogenizing 100 mg of fresh leaf in 1 mL of ice-cold extraction buffer [50 mM potassium phosphate buffer, pH 7.0, containing 4% PVP, 2% glycerol, and 0.1 mM EDTA]. The homogenate was then centrifuged, and the supernatant was used to analyze enzyme activities:

PPO activity was quantified by monitoring purpurogallin formation at 420 nm [51] and calculated using an extinction coefficient of 26.40 M⁻^1^ cm⁻^1^. All activity is expressed as µM g⁻^1^ f.wt. min⁻^1^.

The activity of ascorbate peroxidase (APX) was determined using the assay described by [52]. The reaction was monitored by measuring the change in optical density at 290 nm for 1 min, and the activity was expressed as µM min⁻^1^ g⁻^1^ fresh weight.

### Expression studies

#### Total RNA extraction, cDNA synthesis and RT‑PCR

RNA from Molokhia leaves was extracted utilizing the Qiagen RNase Mini Kit. A thermocycler (MJ Research, Inc., PTC-100TM Programmable Thermal Controller, USA) was utilized to synthesize 20 μl of complementary DNA (cDNA) by RNA reverse transcription. Following one hour of enzyme activation at 42 °C, a five-minute inactivation phase at 95 °C ensued. Conduct triplicate qRT-PCR utilizing Fermentas SYBR Green PCR Master Mix (USA). In each reaction, a 25 μl mixture of primer pairs (*PAL, CAT2, SOD1, FLS*, and *CHLH*) as listed in Table 1 was employed. The reaction was conducted using the RotorGene 6000 (QIAGEN, ABI System, USA). The study utilized the *ACTIN* gene as a reference. The genes analyzed had their relative expression quantified and calculated utilizing the method of [53].

**Table 1:** Specific primers used in this study

Gene name	Abbreviation	Forward (F) and reverse (R) primer 5′–3′
Reference gene	*ACTIN*	F: 5′-GGTAACATTGTGCTCAGTGGTGG-3′ R: 5′-AACGACCTTAATCTTCATGCTGC-3′
Phenylalanine ammonia lyase	*PAL*	F:5′-GCAAGGAAAGCCCGAGTTTAC-3′ R: 5′-GGACCTTTTTGGCTACTTGGC-3′
Flavonol synthase	*FLS*	F: 5′-TTAAAGGAAGGTCTCGGTGGCGAA-3′ R: 5′- TCATTGGTGACGATGAGTGCGAGT −3′
Superoxide dismutase1	*SOD1*	F: 5′-TTCGAAGGGCAGAGTCGAAG-3′ R: 5′-CAAACGAGGCATGCCCAAAA-3′
Catalase2	*CAT2*	F:5′-TGACTGCGGATTCGAGCAA-3′ R: 5′-AACGCAAGAAGAGGGGTTGA-3′
Magnesium chelatase	*CHLH*	F:5′-GGTGAAAATCGTGGAGGAAA-3 R: 5′-ATGTCGCCTCTCAATCCATC-3′

### Statistical analysis

The findings were reported as the means of three replicates, and the standard error (SEs) was computed. One-way ANOVA was employed for statistical analysis to identify significant differences among treatments. Analyses were conducted using XLSTAT software (version 2014.5.03), with a significant threshold was set at Tukey's test, *p* ≤ 0.01.

## Results

### Characterization of MoO_3_NPs

#### UV–visible spectrum of MoO_3_NPs

**Fig. 2:**
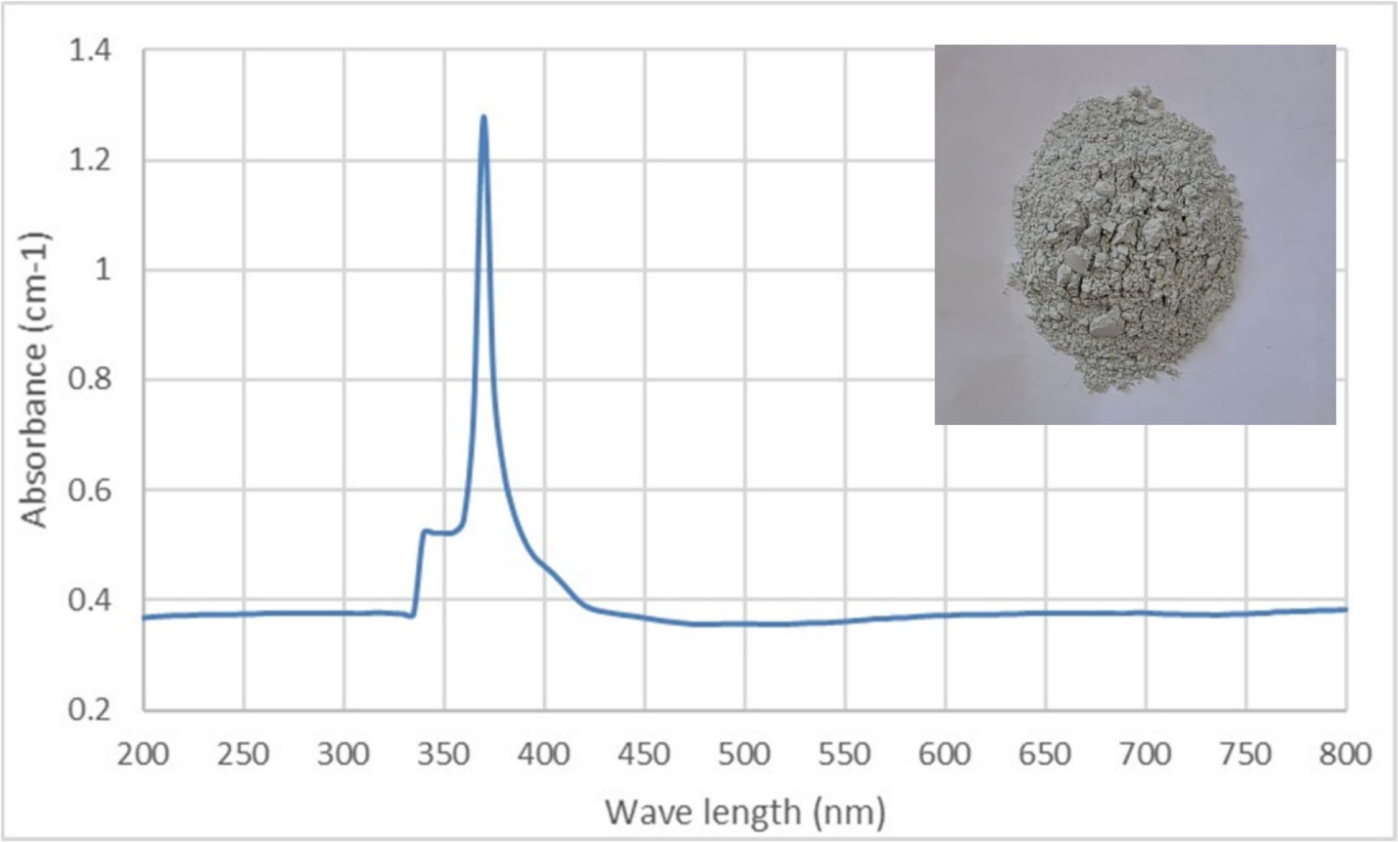
UV–Vis absorption spectrum of biogenically synthesized MoO₃NPs

The biogenic formation of MoO₃NPs, first signaled by a gray color, was confirmed by a UV–Vis spectrum showing a sharp absorption peak at 370 nm within the 200–800 nm range (Fig. 2).

#### Transmission electron micrograph of the synthesized MoO₃NPs

**Fig. 3:**
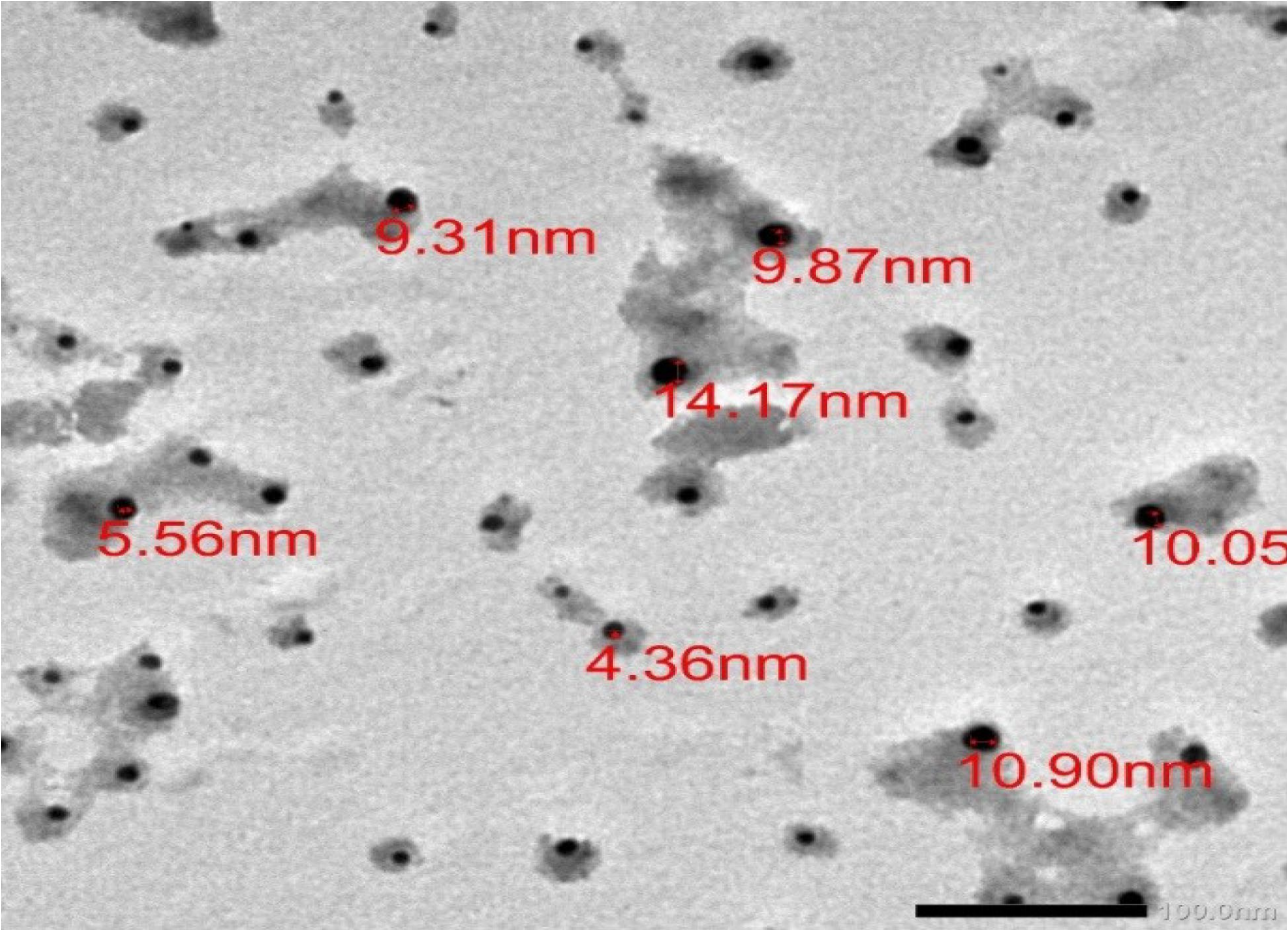
TEM image of biosynthesized MoO_3_NPs

To further characterize the nanoparticles, their size and morphology were examined using transmission electron microscopy (TEM). The TEM micrographs (Fig. 3) confirmed the formation of spherical MoO₃NPs with an average diameter of 9.2 ± 2.7 nm.

#### Fourier transforms infrared spectroscopy of MoO_3_NPs

**Fig. 4:**
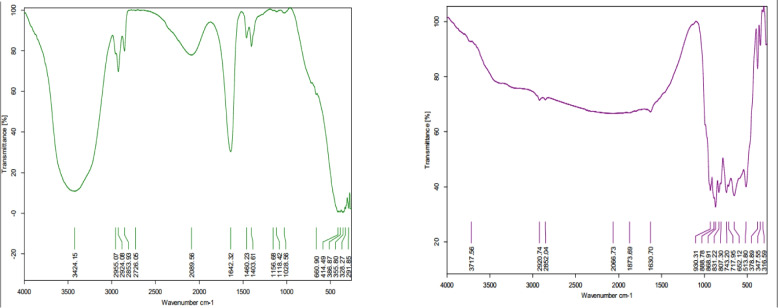
FT-IR analysis of (1) aqueous *M. polymorpha* fruit extract and (2) biosynthesized MoO_3_NPs

To identify the functional groups of the biomolecules involved in the capping and stabilization of the MoO₃NPs, FT-IR analysis was conducted. The resulting spectra (Fig. 4) display transmittance bands across the wavelength range of 400 to 4000 cm-1. The FT-IR analysis of the *M. polymorpha* fruit extract showed distinct absorption peaks at wavenumbers 3424, 2955, 2924, 2853, 2726, 2089, 1642, 1460, and 1403 cm-1. Key peaks from the M. polymorpha extract were also present in the MoO₃NPs FT-IR spectrum (Fig. 4), though slight shifts were noted at 3717, 2920, 2852, 2066, 1873, and 1630 cm⁻^1^. Additionally, the nanoparticle spectrum featured new prominent peaks at lower wavenumbers (930, 888, 831, 807, 743, 717, 652, 513 cm-1). The peak at 513 cm⁻^1^, located within the established inorganic stretch region (400–1000 cm-1), is assigned to the Mo–O stretching vibration, verifying the nanoparticle's composition.

#### X-ray diffraction of MoO_3_NPs

**Fig. 5:**
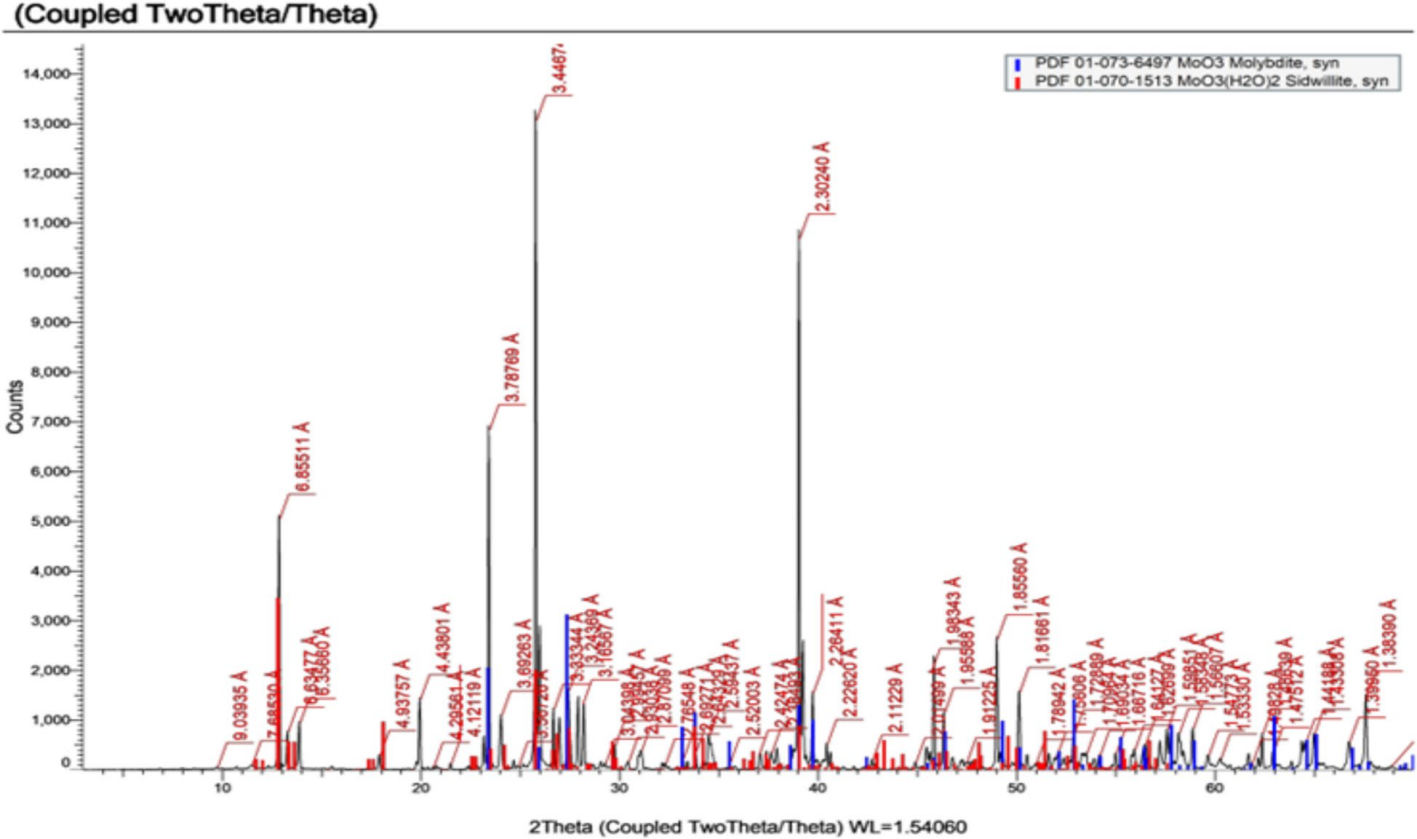
XRD spectra of biosynthesized MoO_3_NPs

The XRD pattern revealed sharp and intense peaks with 2θ values of 23.4°, 25.8°, 27.3° and 39.0° corresponding to the (110), (040), (021), and (060) planes, respectively. These peaks are consistent with the orthorhombic phase of molybdenum trioxide (α‑MoO₃) as defined by JCPDS (Reference code: 01‑073‑6497) as shown in Fig. 5. In addition to the orthorhombic phase, the XRD pattern shows a distinct diffraction peak at a 2θ value of 12.8° which corresponds to the (001) reflection of the hydrated form of MoO₃ using JCPDS (Reference code: 01–070–1513).

### Effect of MoO_3_NPs on some growth parameters

**Fig. 6:**
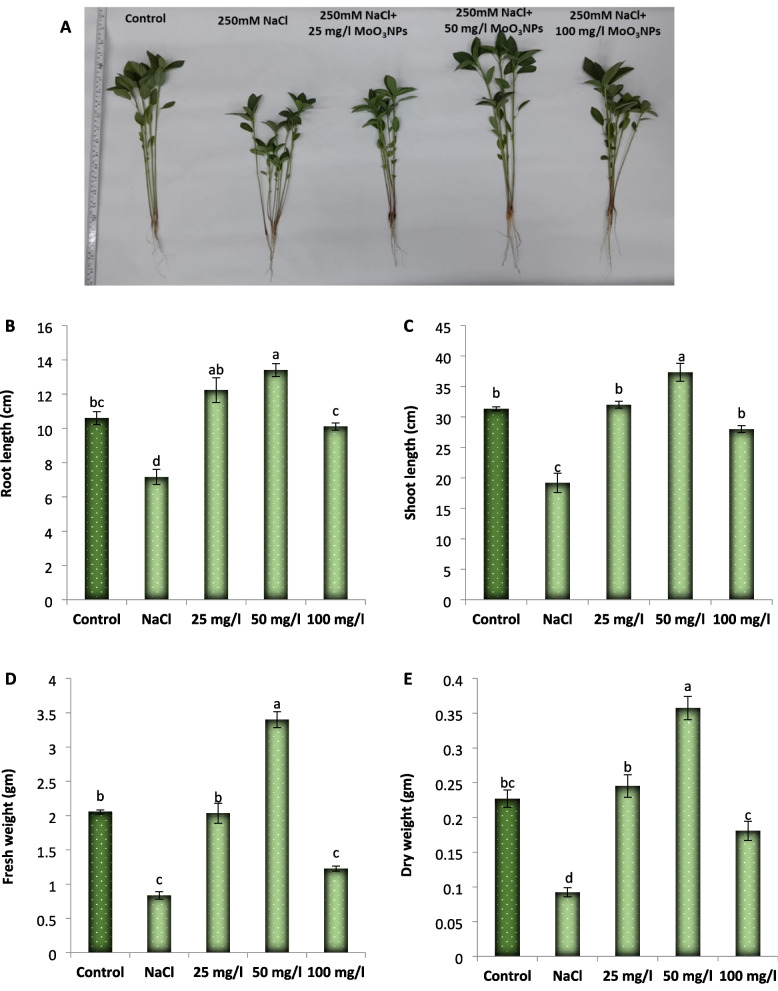
**A** Effect of MoO_3_ NPs foliar spray (0, 25, 50, or 100 mg/L) on the growth parameters of 30-d old salinized* Corchorus olitorius* (Molokhia) plants: **B** Root length, **C** Shoot length, **D** Plant fresh weight, and **E** Plant dry weight. The data represent means ± standard error (n = 3). Different lowercase letters are used to indicate statistically significant differences by Tukey's test at *p* ≤ 0.01

The data represented in Fig. 6 show that the application of 250 mM NaCl led to a notable decrease in (32%), shoot length (39%), fresh weight (59%), and dry weight (59%), relative to those of the control group. Meanwhile, foliar spraying of 25, 50 or 100 mg/L MoO_3_NPs improved all the measured growth parameters of the salinized Molokhia plants. The 50 mg/L dose was the most effective, significantly boosting shoot length by 19.14%, root length by 26.4%, fresh weight by 65.5%, and dry weight by 57.4% relative to the control.

### Effect of MoO_3_NPs on photosynthetic pigments

Application of 250 mM NaCl led to notable decreases in the contents of chlorophyll a, chlorophyll b, and carotenoids and total chlorophyll content of 30-d old Molokhia plant by 25, 11, 23 and 24%, respectively relative to the control groups as represented in Table 2. However, foliar application of MoO₃NPs effectively reversed this decline, significantly boosting pigment concentrations across all treatments. The most substantial enhancement was observed at the 50 mg/L concentration, which increased chlorophyll a, chlorophyll b, and carotenoid levels by 48%, 33% and 67%, respectively, compared to salt-stressed plants.

**Table 2:** Effect of MoO₃NPs foliar spray (0, 25, 50 or 100 mg/L) on pigment content of 30-d old salinized Molokhia plant

Treatment	Chlorophyll a	Chlorophyll b	Carotenoids	Total chlorophyll
Control	1.6 ± 0.03^(ab)^	1.1 ± 0.03^(ab)^	0.6 ± 0.01^(c)^	3.4 ± 0.05^(b)^
250 Mm Nacl	1.2 ± 0.82^(c)^	0.98 ± 0.06^(b)^	0.46 ± 0.008^(d)^	2.6 ± 0.05^(d)^
25 mg/l + 250 Mm Nacl	1.59 ± 0.01^(ab)^	1.2 ± 0.02^(ab)^	0.7 ± 0.006^(b)^	3.49 ± 0.02^(b)^
50 mg/l + 250 Mm Nacl	1.77 ± 0.08^(a)^	1.3 ± 0.12^(a)^	0.77 ± 0.004^(a)^	3.87 ± 0.03^(a)^
100 mg/l + 250 Mm Nacl	1.47 ± 0.03^(ab)^	1.08 ± 0.04^(ab)^	0.57 ± 0.01^(c)^	3.13 ± 0.05^(c)^

### Effect of MoO_3_NPs on oxidative stress

**Fig. 7:**
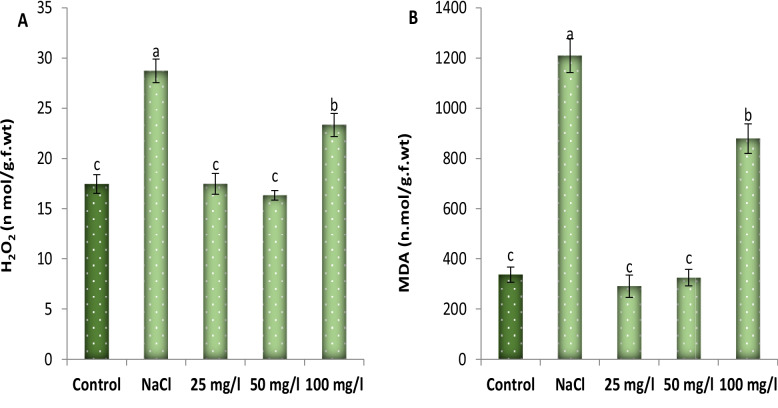
Effect of MoO₃NPs foliar spray (0, 25, 50 or 100 mg/L) on oxidative stress markers ((**A**) MDA and (**B**) H_2_O_2_) of 30-d old salinized Molokhia plant. The data represents means ± standard error (n = 3). Different lowercase letters are used to indicate statistically significant differences at Tukey's test, *p* ≤ 0.01

Application of 250 mM NaCl increased the level of malondialdehyde (MDA) and hydrogen peroxide (H₂O₂) contents of 30-d old Molokhia plants by 259 and 65%, respectively compared with those in the control groups as represented in Fig. 7. Conversely, foliar application of MoO₃NPs significantly suppressed the accumulation of these reactive oxygen species (ROS). The 50 mg/L treatment was particularly effective, achieving a substantial reduction in both H2O2 and MDA levels.

### Effect of MoO_3_NPs on osmolytes

**Fig. 8:**
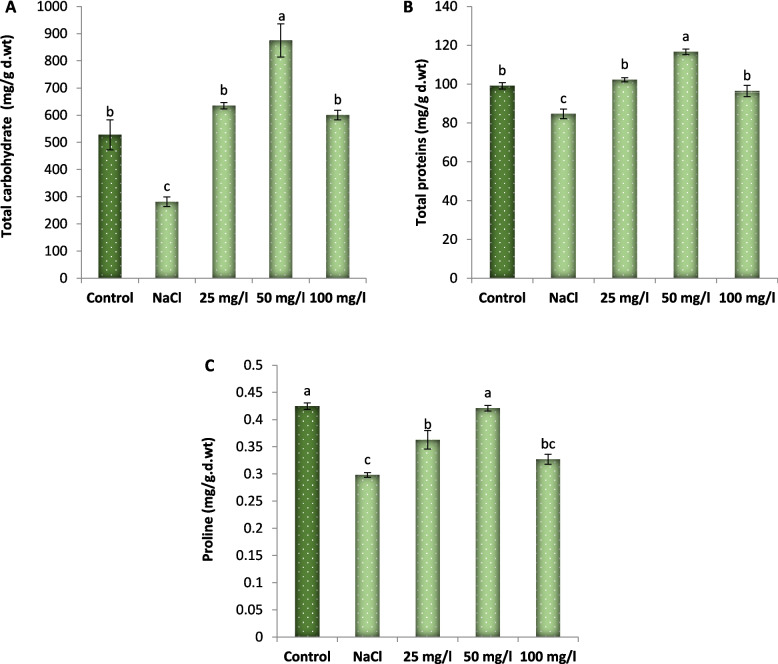
Effect of MoO₃NPs foliar spray (0, 25, 50 or 100 mg/L) on the content of (**A**) total soluble carbohydrates, (**B**) total soluble proteins and (**C**) proline of 30-d old salinized Molokhia plant. The data represents means ± standard error (*n* = 3). Different lowercase letters are used to indicate statistically significant differences at Tukey's test, *p* ≤ 0.01

The present investigation revealed that treating Molokhia plants with 250 mM NaCl significantly diminished the content of total soluble carbohydrates, total soluble proteins and proline of 30-d old Molokhia plant by approximately 47, 15 and 31%, respectively in comparison with the control samples as represented in Fig. 8. Meanwhile, foliar spraying with 25, 50 or 100 mg/L MoO_3_NPs had a more pronounced effect on increasing the content of total soluble carbohydrates, total soluble proteins and proline of salinized Molokhia plant*.* Compared with the salt-stressed plants, treatment with 50 mg/l SNPs was the most effective and increased the soluble sugar content by 211%, total soluble proteins content by 38%, and proline content by 45%.

### Effect of MoO_3_NPs on non-enzymatic and enzymatic antioxidants

#### Non-enzymatic antioxidants

**Fig. 9:**
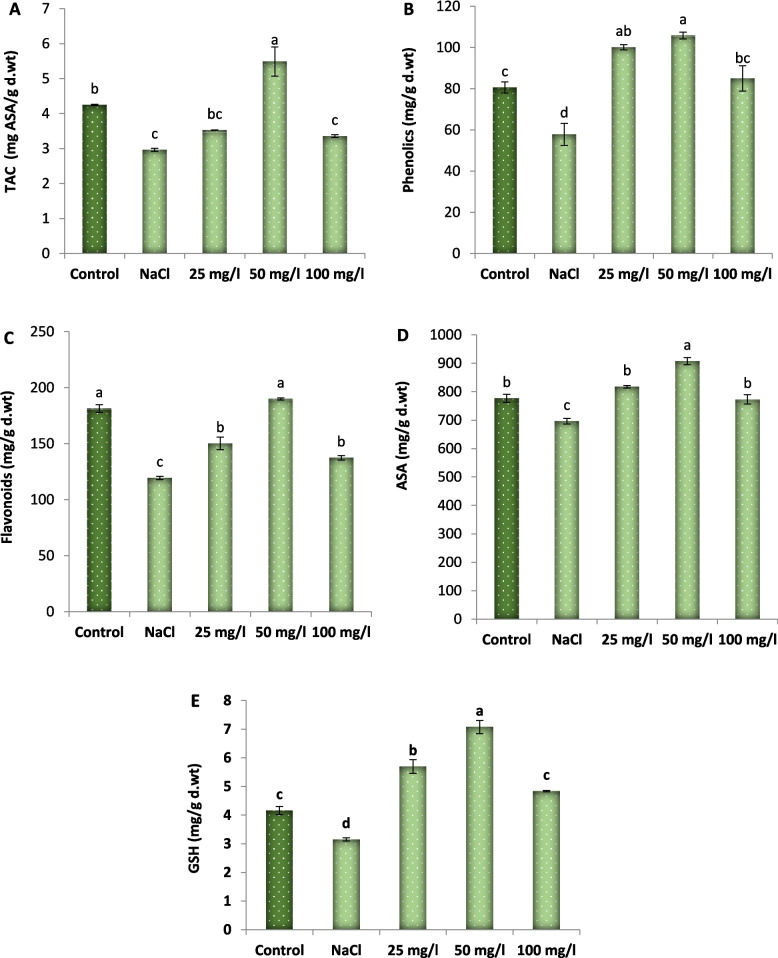
Effect of MoO₃NPs foliar spray (0, 25, 50 or 100 mg/L) on non-enzymatic antioxidants ((**A**) TAC, (**B**) total phenolic, (**C**) total flavonoids, (**D**) ASA, and (E) GSH) of 30-d old salinized Molokhia plant. The data represents means ± standard error (*n* = 3). Different lowercase letters are used to indicate statistically significant differences at Tukey's test, *p* ≤ 0.01

As shown in Fig. 9 the content of total antioxidant capacity (TAC), total phenolic, total flavonoids, ascorbic acid (ASA), and reduced glutathione (GSH) of 30-d old Molokhia plants were significantly diminished, compared to control plants, by 31, 28, 34, 10, and 24%, respectively due to application of 250 mM NaCl. However, MoO_3_NPs application markedly enhanced the activity of all assayed non-enzymatic antioxidants in the salinized Molokhia plants. The 50 mg/L concentration demonstrated the highest efficacy; the ratios of improvement were 86% for TAC; 83% for total phenolic, 59% for total flavonoids, 30% for ASA, and 121% for GSH.

#### Enzymatic antioxidants

**Fig. 10:**
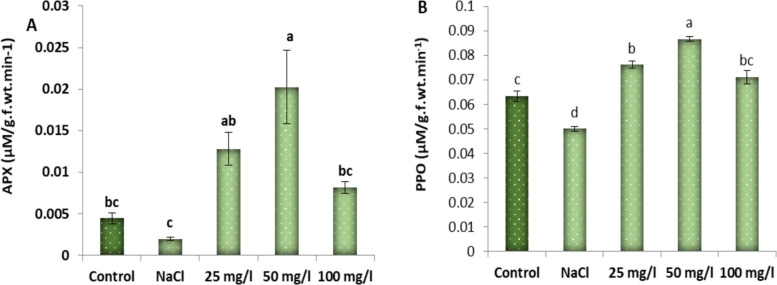
Effect of MoO₃NPs foliar spray (0, 25, 50 or 100 mg/L) on the activity of (**A**) PPO and (**B**) APX of 30-d old salinized Molokhia plant. The data represents means ± standard error (*n* = 3). Different lowercase letters are used to indicate statistically significant differences at Tukey's test, *p* ≤ 0.01

The results represented in Fig. 10 show that treating Molokhia plants with 250 mM NaCl significantly diminished the activity of polyphenol oxidase (PPO) and ascorbate peroxidase (APX) of 30-d old Molokhia plants by 17 and 57%, respectively, relative to the control groups. Meanwhile, foliar spraying of 50 mg/L MoO_3_NPs was the highest improvement in the activity of PPO by 0.086 ± 0.001 µM/g.f.wt.min-1, relative to salt-stressed plants (0.05 ± 0.002 µM/g.f.wt.min-1). Additionally, APX activity boosted from 0.0019 ± 0.0001µM/g.f.wt.min-1 (in salinized Molokhia plants) to 0.0202 ± 0.004 µM/g.f.wt.min-1.

### Gene expression

**Fig. 11:**
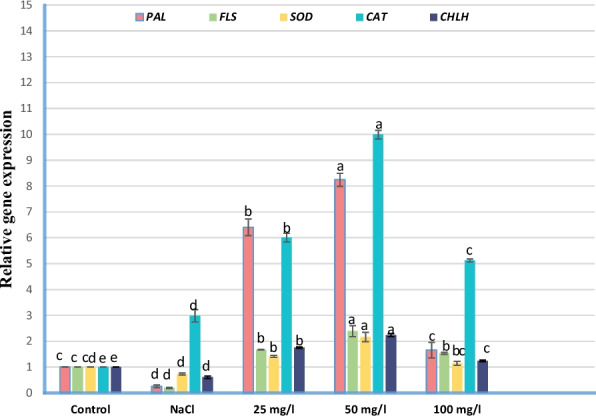
Effect of MoO₃NPs foliar spray (0, 25, 50 or 100 mg/L) on the relative gene expression of *PAL*, *SOD*, *CAT*, *FLS*, and *CHLH* in 30-d old salinized Molokhia plants. The data represents means ± standard error (*n* = 3). Different lowercase letters are used to indicate statistically significant differences at Tukey's test, *p* ≤ 0.01

Exposure to severe salinity stress (250 mM NaCl) induced a significant downregulation in the transcript levels of *PAL, FLS, SOD1*, and *CHLH* compared to the unstressed control plants. In contrast, *CAT2* expression was up-regulated under saline conditions, suggesting a compensatory mechanism to mitigate hydrogen peroxide accumulation. The application of MoO₃NPs effectively triggered a dose-dependent recovery response. Specifically, the 50 mg/L MoO₃NPs treatment proved most effective, resulting in a robust upregulation of all studied genes. Compared to the non-stressed treatment, this dose increased the expression levels of *PAL, SOD1, CAT2, FLS,* and *CHLH* by 0.6, 1.16, 8.9, 1.4, and 1.2 fold, respectively. These results indicate that MoO₃NPs enhance salt tolerance by stimulating the phenylpropanoid pathway and enzymatic antioxidant system, thereby maintaining cellular redox homeostasis and photosynthetic integrity under stress (Fig. 11).

### Pearson correlation coefficient

**Fig. 12:**
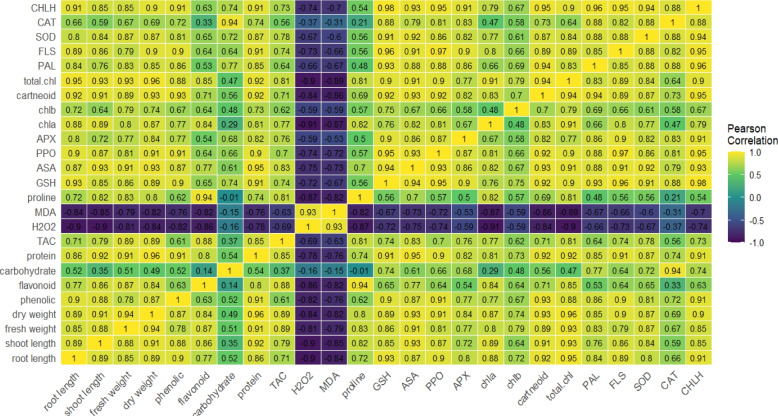
Correlation heatmap illustrating the relationships between morphological, physiological, and molecular parameters. The analysis was based on three biological replicates per treatment. Treatments included Molokhia plants treated with 25, 50 and 100 mg/L MoO_3_NPs under control or 250mM NaCl (salinity stress)

Pearson's simple correlation provided a comprehensive visualization of the interrelationships among physiological, biochemical traits and molecular approaches in plants observed in various treatments with biogenic MoO_3_NPs applications (Fig. 12). All growth metrics (root length, shoot length, fresh weight, dry weight) exhibited significant positive correlations with photosynthetic pigments (chlorophyll a, chlorophyll b, total chlorophyll, and carotenoids), carbohydrate and soluble proteins. Also, *CHLH* gene exhibits very strong positive correlations with the core components of photosynthesis and growth metrics. Moreover, it is strongly correlated with the antioxidant system, including GSH and ASA, suggesting a coordinated response where maintained chlorophyll biosynthesis is supported by a robust antioxidant capacity to mitigate oxidative damage. Furthermore, TAC, ASA, and GSH were highly intercorrelated and also showed strong positive links to the enzymatic antioxidants (*SOD1*, *CAT2*, and APX) and with improved growth metrics. Furthermore, PAL and FLS expression was strongly positively correlated with the phenolic compounds and flavonoids. The entire antioxidant system (enzymatic, non-enzymatic, and phenylpropanoid-derived compounds) was positively correlated with improved growth parameters. The oxidative stress markers MDA and H₂O₂ were strongly positively correlated with each other. As expected, both markers were strongly negatively correlated with growth metrics and components of the antioxidant system. Proline content showed a very strong positive correlation with flavonoid content.

### Principal component analysis (PCA)

**Fig. 13:**
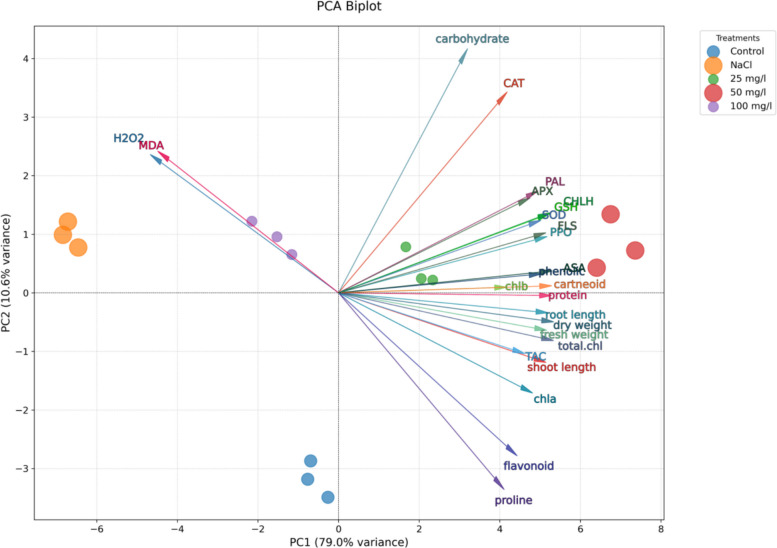
Principal component analysis (PCA) of Molokhia plant growth, genetic, and physiological parameters at varying concentrations of MoO_3_NPs (0, 25, 50 or 100 mg/L) in 30-d old salinized Molokhia plants

**Fig. 14:**
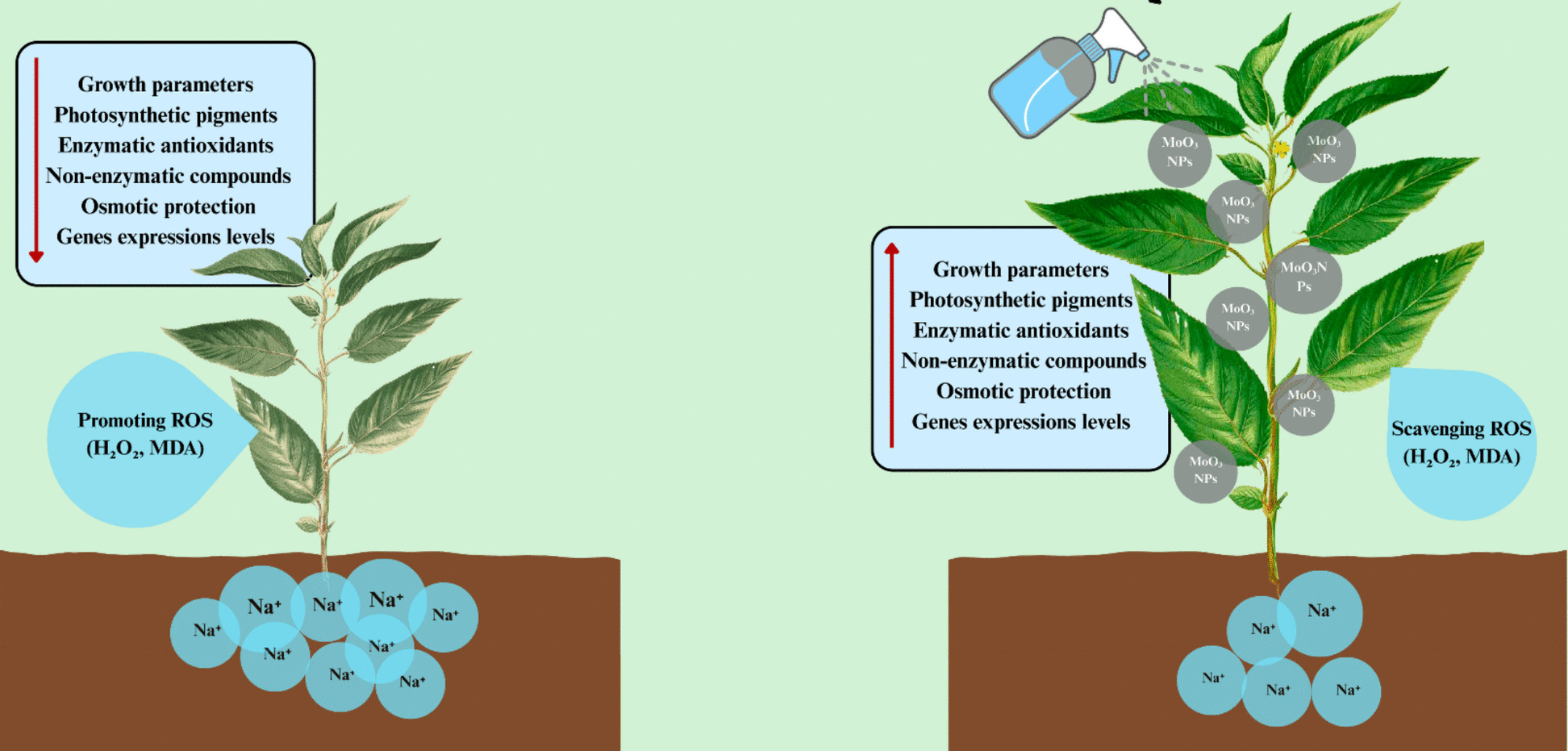
MoO_3_ nanoparticle treatment enhances plant growth and reduces oxidative stress and sodium accumulation

The Principal Component Analysis (PCA) successfully differentiated the treatment groups and revealed key patterns in the response of Molokhia plants to salt stress and MoO₃NP treatment (Fig. 13). The first principal component (PC1) captured the overwhelming majority of the variance (79%), effectively separating the treatments along a primary gradient of stress response and recovery. The control group and the plants treated with 50 mg/l MoO₃NPs clustered on the positive side of PC1. Their positioning was strongly associated with vectors for growth traits, photosynthetic pigments, osmolytes, non-enzymatic antioxidants (ASA, GSH, TAC, and phenolic), and enzymatic antioxidants (*SOD*, APX, PPO, *CHLH*, and *PAL*). This grouping reflects enhanced growth, pigment stability, and antioxidant defense under these treatments. The 25 mg/l MoO₃NP treatment group was positioned between the NaCl group and the healthier clusters, and was closer to the center of the biplot. This suggests that this concentration provided a partial mitigation effect, moving the plants away from the severe stress profile but not fully restoring them to the non-stressed state achieved by the higher nanoparticle concentrations. Conversely, the NaCl-stressed plants and 100 mg/l MoO₃NPs were positioned at the far positive side of PC1, closely aligned with oxidative stress markers (MDA, H₂O₂), indicating high oxidative damage and a stress response (Fig. 14).

## Discussion

Salinity stress, as a global problem, causes impairment in plant growth via disrupting many physiological and biochemical processes. Nano-fertilizers offer a superior alternative to chemical fertilizers by significantly improving resource use efficiency and crop yields, while also reducing environmental pollution as recommended by [54]. In addition, green synthesized nano-particles can be regarded as powerful, inexpensive nano-fertilizers that can replace conventional products, thereby solving the problem of excessive agrochemical application [55, 56]. Therefore, we biosynthesized MoO_3_NPs using an extract from *Medicago polymorpha* fruit, which was then used to evaluate the effectiveness of MoO_3_NPs to ameliorate the Molokhia plant's resistance to salt stress. The biosynthesized MoO_3_NPs powder appeared gray color and was characterized by using UV–Visible spectroscopy. The resulting UV–visible spectrum indicated that the MoO_3_NPs had a peak at 370 nm in UV region. These results are in agreement with those obtained by [57, 58], who reported that MoO_3_NPs have absorption spectra between 321 and 398 nm. Additionally, TEM confirmed the formation of spherical MoO₃NPs with possessed a narrow size distribution, averaging 9.2 ± 2.7 nm. For further analysis, the XRD pattern was consistent with the orthorhombic crystalline phase of molybdenum trioxide (α-MoO₃), which is consistent with the results reported by [59].

The stretching O–H peak appears in *M. polymorpha* fruit extract spectrum as a very distinctive band extends from peak recorded at 3424 cm⁻^1^ (alcohols, carboxylic acids, phenols). A shift observed in this band for the synthesized MoO₃NPs is likely caused by the attachment of molybdenum ions to hydrogen atoms (Shafieyan et al. 2019). The stretching C-H appears at 2955–2853 cm-1 (alkanes), and signals for aldehydes (2726 cm-1), alkynes (2089 cm-1), and C = N/C = C (1642 cm-1) appear. These signify a substantial presence of phenolic chemicals, flavonoids, and aliphatic constituents. The detected peak shifts in biogenic MoO_3_NPs, specifically the flavonoid-related peak at 1642 cm-1 and alkane-related peak at 2955–2853 cm-1, suggest that the organic molecules from the fruit extract functioned as reducing and stabilizing agents, resulting in the formation of an organic–inorganic hybrid structure. For the MoO_3_NPs, the prominent peaks were observed in the FT-IR spectrum at 930, 888, 831, 807, 743, 717, 652, and 513 cm-1. The peaks at 930 cm-1 and 888 cm-1 indicative of organic capping agents, exhibited slight alterations in the MoO_3_NP, implying their contact with the nanoparticle surface. The significant peak at 513 cm⁻^1^ unambiguously confirms the stretching of MoO₃, hence validating the production of nanoparticles. This finding validates the effectiveness of *M. polymorpha* in the green manufacturing of MoO_3_NP, with the fruit extract components successfully generating and coating the nanoparticles. Similar absorption peaks were recorded by [60], emphasizing analogous alterations in functional groups during the environmentally benign manufacturing of MoO_3_NP.

In our present investigation, the application of 250 mM NaCl led to a marked decrease in growth parameters (including root length, shoot length, dry weight and fresh weight) of 30-d old Molokhia plants. This reduction may result from a disturbed water balance and metabolic activities [61], alongside restricted cell elongation and division that inhibit physiological and biochemical processes [62]. Additionally, compromised uptake of essential nutrients could explain the decline in biomass recorded in the salt-stressed plants [63]. Meanwhile, foliar spraying of MoO_3_NPs improved all the measured growth parameters of the salinized Molokhia plants, particularly at a concentration of 50 mg/l. These findings align with those of previous studies demonstrating the beneficial effects of molybdenum nanoparticles (MoNPs) on the growth of rice, tobacco, and chickpea [64–66]. Molybdenum (Mo), a crucial trace element, acts as an essential cofactor for nitrate reductase in plant nitrogen metabolism, playing an indispensable role in growth and stress response [27]. Since molybdenum plays a key role in improving nutrient uptake and maintaining ionic balance [67], it can activate a variety of physiological signaling pathways which in turn overcomes the deleterious effects of salt stress on the development and metabolism of Molokhia plants. It has been demonstrated that MoO_3_NPs enhance the synthesis of indole-3-acetic acid and improve phosphate solubilization, both of which are critical processes for plant growth and stress tolerance [68].

Data of present investigation showed how application of 250 mM NaCl causes the decreased contents of chlorophyll a, chlorophyll b, carotenoids and total chlorophyll content of 30-d old Molokhia plants. Salinity stress reduced chlorophyll content in Molokhia leaves, likely due to inhibited chlorophyll biosynthesis, nutrient deficiency, increased chlorophyllase activity, reduced membrane stability, and excessive Na⁺ accumulation disrupting photosynthetic pigments [69]. This notable reduction in photosynthetic pigments caused by salt stress was successfully counteracted by foliar spraying of MoO_3_NPs, particularly at a concentration of 50 mg/l. These findings may be attributed to the ability of molybdium to enhance photosynthesis rates through the enhancement of non-stomatal factors, maintaining ionic balance by increasing Ca⁺/Na⁺, K⁺/Na⁺, and Mg⁺/Na⁺ ratios [70]. According to [66], Mo NPs likely enhance chlorophyll levels in tobacco by supplying molybdenum, an essential element for key metabolic enzymes, thus promoting chloroplast development and chlorophyll synthesis. Similar to MoO₃NPs, the activated mechanisms may involve the upregulation of the *CHLH* gene. The dramatic reduction of CHLH expression under 250 mM NaCl explains the observed chlorosis in salinized plants. The ability of MoO₃NPs to restore *CHLH* transcript levels is a critical finding. As CHLH is a subunit of Mg-chelatase and a known ABA receptor, its upregulation is indicative of a potential dual response where MoO₃NPs may help maintain Mg-insertion into the porphyrin ring for chlorophyll synthesis [71], and modulating ABA-mediated stomatal signaling to prevent excessive water loss [71, 72]. This retrograde signaling role of CHLH ensures that the chloroplast can communicate its functional status to the nucleus, maintaining a coordinated stress response. This result was confirmed by the Pearson correlation coefficient (Fig. 12) that recorded *CHLH* gene exhibits very strong positive correlations with the core components of photosynthesis and antioxidant system, suggesting that a coordinated response where maintained chlorophyll biosynthesis is supported by a robust antioxidant capacity to mitigate oxidative damage.

The imposition of salinity stress induces stomatal closure, thereby restricting the availability of CO_2_ within the leaf and suppressing carbon fixation. Consequently, chloroplasts are subjected to excess excitation energy, leading to an elevated production of ROS, namely, H₂O₂, superoxide, and hydroxyl radicals [73]. The excessive production of ROS, in response to various environmental stresses, leads to membrane disfunction and causes cell death [74]. The present study's findings demonstrated the elevated MDAand H₂O₂ contents of 30-d old Molokhia plant in response to application of 250 mMNaCl. This refers to major lipid peroxidation and redox imbalance resulting from the accumulation of ROSwhich damages cellular integrity [75]. Furthermore, foliar spraying of MoO_3_NPs diminished the content of oxidative stress markers (MDA and H_2_O_2_) in salinized Molokhia plants, which highlights the effectiveness of MoO_3_NPs in improving cellular absorption and intracellular mobility and mitigating oxidative damage via ROS detoxification [76]. Additionally, molybdenum application also alters the fatty acid composition of thylakoid membranes, increasing the ratio of unsaturated to saturated fatty acids, leading to improved membrane fluidity and stability in wheat [77].

Osmotic adjustment is a fundamental physiological strategy employed by higher plants to address environmental constraints, including sugars, total soluble protein, and proline [78]. In our work, salinity stress induced a significant reduction in the content of total soluble carbohydrates, total soluble proteins and proline of 30-d old Molokhia plant. Salinity stress suppressed carbohydrate and protein levels, which aligns with [79], who attributed such decline to osmotic stress-induced reductions in root hydraulic conductivity and cell volume, impairing nutrient/water flow and metabolic functions like protein synthesis. Additionally [80], reported no proline increase in select *Sorghum bicolor* accessions under salinity stress. However, foliar spraying of MoO_3_NPs boosted osmolyte levels of the salinized Molokhia plant, particularly at concentrations of 50 mg/L. MoO_3_NPs have proven effective in enhancing osmolyte production and stress responses. In the same manner, [66] showed MoNPs elevated soluble sugars and antioxidants in tobacco. Elevated proline within plant cells serves a vital osmoregulatory function under stress conditions, which stabilizes macromolecules and cellular membranes to confer greater abiotic stress tolerance [81]. According to [82], the application of foliar cerium oxide nanoparticles (CeO₂NPs) elevated osmolyte levels in salt-stressed *Dracocephalum moldavica* L. Notably, proline, a key osmolyte cited in their study, accumulates to regulate cellular osmotic potential and minimize water loss, a mechanism that enhances salinity tolerance.

As salinity stress can induce oxidative stress via overproduction of ROSwhich leading to cellular impairment [83]. The current study's results show that application of 250 mMNaCl diminished both enzymatic and non-enzymatic antioxidants (including TAC, flavonoids, phenolic, ASA, GSH, APX, and PPO) and relative gene expression of *PAL, FLS,* and *SOD* in 30-d old Molokhia plants. However, foliar spraying of MoO_3_NPs improved all assayed enzymatic and non-enzymatic antioxidants of the salinized Molokhia plant, particularly at 50 mg/l MoO_3_NP. The first pathway of the defense system against the oxidative damage is SOD [84]. The increased activity of SOD is critical in converting O_2_^•−^ to H_2_O_2_ which is consequently detoxified thru APX and CAT [85]. The enhanced relative gene expression of *SOD1* and *CAT2* could support the hypothesis that H_2_O_2_ results from oxygen free radicals including O_2_^•−^ [86, 87]. This aligns with findings in rice and wheat where metal oxides nanoparticles such as ZnO and CuO were shown to modulate the expression of *SOD* and *CAT* isoforms, effectively scavenging O_2_^•−^ and H_2_O_2_ before they could induce lipid peroxidation [88, 89]. Interestingly, the increase in *CAT2* expression in our study suggests that MoO_3_NPs may specifically target the peroxisomal detoxification pathway, which is often the first to be compromised under high salinity (250 mM NaCl). According to reports, plants become more resilient to oxidative stress when the majority of *SOD1* and *CAT2* genes are over expressed [55]. Alongside, ascorbic acid and glutathione have been reported to improve osmoregulation, water use efficacy, photosynthetic activity, and then the overall productivity of plants. The ASA-GSH cycle is responsible for detoxification of H_2_O_2_ by stimulating APX activity [90]. Additionally, ASA, *CAT2*, *SOD1*, PPO, APX, TAC, POD, and GSH, were strongly correlated according to Pearson correlation coefficient (Fig. 12). These results confirm that antioxidants, both enzymes like SOD and CAT, and compounds like ASA, GSHand flavonoids, work as a team to keep ROS levels in check and stop them from damaging the cell, which supports the conclusions of [91].

In addition to what was mentioned above, PAL is a pivotal inducible enzyme in the phenylpropanoid pathway. It catalyzes the initial step by deaminating L-phenylalanine to form trans-cinnamic acid, serving as a gateway for the biosynthesis of critical phenolic compounds like lignin, flavonoids, and pigments [92, 93]. Meanwhile, FLS is a critical enzyme responsible for flavonol production [94]. These compounds, which are part of the broader phenolic and flavonoid groups, are vital for mediating plant biological interactions and enhancing stress resilience. This accumulation is often driven by the upregulation of pivotal biosynthetic enzymes like PAL, with studies confirming a strong positive correlation between PAL activity and phenolic content [95]. The de novo synthesis of phenolics and flavonoids, driven by PAL, serves as a secondary line of defense against salinity stress. Similar transcriptional activations of the phenylpropanoid pathway (*PAL, FLS*) have been reported in Wheel wingnut plants treated with SiO_2_NPs where the nanoparticles act as elicitors that trigger the expression of stress-responsive secondary metabolites [96]. By up-regulating *FLS*, MoO_3_NPs likely promote the accumulation of quercetin-type flavonoids, which are potent hydroxyl radical scavengers [97], thereby protecting the cellular integrity of Molokhia. Research by [55, 56] indicates that treating plants with biogenic nanoparticles under stress significantly boosts the activity of key antioxidants, both enzymatic and non-enzymatic. This study offers a novel perspective on biogenic MoO_3_NPs in saline agriculture, revealing for the first time their role in transcriptionally reprogramming the antioxidant and biosynthetic pathways of Molokhia. The strong correlation between the up-regulation of *PAL, SOD1, CAT2, FLS,* and *CHLH* and improved growth metrics provides a molecular blueprint for MoO_3_NPs mediated salt tolerance. These findings establish molybdenum-based nanobiotechnology as a sustainable strategy for reclaiming saline lands and securing the production of salt-sensitive leafy vegetables.

## Conclusion

In conclusion, this study successfully demonstrates the efficacy of green-synthesized molybdenum trioxide nanoparticles (MoO₃NPs) in mitigating the detrimental effects of salinity stress on Molokhia plants. The research unequivocally shows that salinity stress (250 mM NaCl) severely impairs plant growth, photosynthetic pigment synthesis, osmotic balance, and antioxidant defense systems, while inducing significant oxidative damage. The foliar application of MoO₃NPs, particularly at 50 mg/L, effectively reversed these adverse effects. The nanoparticles enhanced growth parameters, restored chlorophyll content, improved osmotic adjustment through increased proline and soluble solutes, and most critically, bolstered the plant's antioxidant machinery. Therefore, the green synthesis of MoO₃NPs presents a powerful, sustainable, and eco-friendly nano-agricultural strategy to enhance crop resilience and productivity in salinity-affected soils, offering a promising alternative to traditional fertilization methods. From an agronomic perspective, the biogenic synthesis of MoO_3_NPs offers a low-cost and sustainable alternative to traditional mineral fertilizers. However, while the synthesis phase is economically favorable, the precise cost–benefit ratio and environmental footprint of repeated field-scale applications remain to be fully quantified in future long-term studies.

## Data Availability

All data supporting the findings of this study are already presented in this published manuscript.

## Abbreviations

MoO₃NPs: Molybdenum trioxide nanoparticles
XRD: X-ray diffractometer
TEM: Transmission electron microscopy
FTIR: Fourier Transform Infrared Spectroscopy
APX: Ascorbate peroxidase
PPO: Polyphenol oxidase
*PAL*: *phenylalanine*
*CAT2*: *catalase2*
*CHLH*: *magnesium chelatase*
*SOD1*: *superoxide dismutase1*
*FLS*: *flavonol synthase*
TAC: Total antioxidant capacity
ASA: Ascorbic acid content
H_2_O_2_: Hydrogen peroxide
MDA: Malondialdehyde
PCA: Principle component analysis
ANOVA: Analysis of Variance
